# Supplementary material for: COVID-19-Associated Pulmonary Aspergillosis in Intensive Care Unit Patients from Poland
Source: J Fungi (Basel). 2023 Jun 13;9(6):666. doi: 10.3390/jof9060666 (PMC10303628; doi:10.3390/jof9060666)
Supplement: Supplementary file 1 [file jof-09-00666-s001.zip › jof-2412694-supplementary.pdf]

Supplementary Information (SI)

COVID-19-Associated Pulmonary Aspergillosis in Intensive Care Unit Patients from Poland

Magdalena Skóra [1,*], Mateusz Gajda [1], Magdalena Namysł [2], Jerzy Wordliczek [3], Joanna Zorska [4,5], Piotr Piekiełko [6,7], Barbara Żółtowska [8], Paweł Krzyściak [1], Piotr B. Heczko [1] and Jadwiga Wójkowska-Mach [1]

1. Chair of Microbiology, Faculty of Medicine, Jagiellonian University Medical College, Czysta 18 Street, 31-121 Krakow, Poland; mateusz14.gajda@uj.edu.pl (M.G.); pawel.krzysciak@uj.edu.pl (P.K.); piotr.heczko@uj.edu.pl (P.B.H.); jadwiga.wojkowska-mach@uj.edu.pl (J.W.-M.)
2. Department of Microbiology, University Hospital in Krakow, Macieja Jakubowskiego 2 Street, 30-688 Krakow, Poland; mnamysl@su.krakow.pl
3. Interdisciplinary Intensive Care Clinic, Jagiellonian University Medical College, Macieja Jakubowskiego 2 Street, 30-688 Krakow, Poland; j.wordliczek@uj.edu.pl
4. Center for Innovative Medical Education, Jagiellonian University Medical College, Medyczna 7 Street, 30-688 Krakow, Poland; jzorska@su.krakow.pl
5. Intensive Care Unit, University Hospital in Krakow, Macieja Jakubowskiego 2 Street, 30-688 Krakow, Poland
6. Department of Internal Diseases and Circulatory Failure, Center of Pulmonology and Thoracic Surgery in Bystra, Juliana Fałata 2 Street, 43-360 Bystra, Poland; ppiekielko@szpitalbystra.pl
7. Department of Pulmonology and Respiratory Failure, Center of Pulmonology and Thoracic Surgery in Bystra, Juliana Fałata 2 Street, 43-360 Bystra, Poland
8. Center for Innovative Therapy, Clinical Research Coordination Center, University Hospital in Krakow, Macieja Jakubowskiego 2 Street, 30-688 Krakow, Poland; bzoltowska@su.krakow.pl

* Correspondence: magdalena.skora@uj.edu.pl

Journal name: Journal of Fungi

Table S1. Characteristics of patients with CAPA

**Patient no**	**Patient sex/age**	**Starting of hospitalisation in the ICU**	**Underlying diseases**	**APACHE II at ICU admission**	**SAPS II at ICU admission**	**Immunosuppresion before Covid-19**	**Clinical features**	**Radiological findings**	**Invasive mechanical ventilation**	**Corticosteroid theraphy**	**Antibiotic theraphy**	**Antifungal theraphy**	**Outcome**
57	F/33	28.05.2021	pneumonia, pneumothorax	25	56	no	dyspnea, respiratory insufficiency despite ventilation support	CT: nonspecific infltrates and consolidation, crazy paving sign	28.05.2021	dexamethasone (29.05.2021)	ceftriaxone (23.05.2021) levofloxacin (23.05.2021) linezolid (02.06.2021) colistin (04.06.2021) meropenem (04.06.2021)	voriconazole (16.06.2021)	death 17.06.2021 (after transfer to another ICU)
104	M/60	15.09.2021	asthma	23	74	no	dyspnea, respiratory insufficiency despite ventilation support	CT: dense, well-circumscribed lesions with halo sign, diffuse reticular and alveolar opacities,	15.09.2021	dexamethasone (16.09.2021)	meropenem (15.09.2021)	voriconazole (22.09.2021)	death 24.09.2021

								nonspecific inflltrates and consolidation, pleural fluid, crazy paving sign					
132	M/67	30.10.2021	diabetes mellitus, fatty liver disease, hypercholesterolemia, atherosclerosis, double kidney on left with enlargement, gastritis, pancreatitis	20	38	no	fever refractory to > 3 days of antibiotherapy, dyspnea, respiratory insufficiency despite ventilation support	CT: diffuse reticular and alveolar opacities, nonspecific inflltrates and consolidation, crazy paving sign	30.10.2021	dexamethasone (30.10.2021)	levofloxacin (31.10.2021) ceftriaxone (02.11.2021) colistin (07.11.2021) meropenem (11.11.2021) linezolid (11.11.2021)	none	alive (discharge 08.12.2021)
150	M/58	31.10.2021	obesity, anaemia, chronic heart failure, ulcerative disease, alcoholic hepatic cirrhosis	25	64	no	dyspnea, respiratory insufficiency despite ventilation support	X-ray: diffuse reticular and alveolar opacities, nonspecific inflltrates and consolidation,	01.11.2021	dexamethasone (31.10.2021)	rifaximin (31.10.2021) levofloxacin (31.10.2021) ceftriaxone	none	death 04.11.2021

								suspicion of pleural fluid			(31.10.2021) meropenem (04.11.2021)		
181	M/66	22.11.2021	none	11	33	no	fever refractory to > 3 days of antibiotherapy, dyspnea, respiratory insufficiency despite ventilation support	CT: nonspecific infltrates and consolidation, crazy paving sign	25.11.2021	dexamethasone (23.11.2021)	ceftriaxone (22.11.2021) meropenem (28.11.2021) linezolid (28.11.2021)	none	death 30.11.2021
182	M/60	21.11.2021	none	32	55	no	dyspnea, respiratory insufficiency despite ventilation support	X-ray: nonspecific infltrates and consolidation	21.11.2021	dexamethasone (22.11.2021)	ampicillin + sulbactam (27.11.2021) meropenem (28.11.2021) linezolid (28.11.2021) amikacin (01.12.2021)	fluconazole (30.11.2021)	death 06.12.2021
187	M/78	22.11.2021	heart failure	47	88	no	fever refractory to > 3 days of	CT: cavity, diffuse reticular	22.11.2021	dexamethasone (22.11.2021)	linezolid (22.11.2021)	fluconazole (30.11.2021)	death 06.12.2021

							antibiotherapy, dyspnea, respiratory insufficiency despite ventilation support	and alveolar opacities, nonspecific infltrates and consolidation, crazy paving sign			meropenem (22.11.2021) amikacin (03.12.2021)		
190	F/56	28.11.2021	hyperlipidemia	27	61	no	dyspnea, respiratory insufficiency despite ventilation support	CT: dense, well-circumscribed lesions with halo sign, nonspecific infltrates and consolidation, crazy paving sign	28.11.2021	dexamethasone (29.11.2021)	none	voriconazole (03.12.2021)	alive (discharge 11.01.2022)
204	F/60	03.12.2021	lupus with nephritis, anaemia, leukopenia, hypertension, heart failure, fatty liver disease	32	55	yes (corticosteroids > 0.3 mg/kg/day for > 3 weeks, methotrexate therapy)	dyspnea, respiratory insufficiency despite ventilation support	CT: dense, well-circumscribed lesions with halo sign, nonspecific infltrates and consolidation, crazy paving	03.12.2021	dexamethasone (04.12.2021)	vancomycin (08.12.2021) linezolid (11.12.2021) meropenem (11.12.2021)	none	death 12.12.2021

								sign, pleural fluid					
214	F/75	17.12.2021	none	27	56	no	fever refractory to > 3 days of antibiotherapy, dyspnea, respiratory insufficiency despite ventilation support	CT: nonspecific infltrates and consolidation, crazy paving sing, honeycombing	17.12.2021	dexamethasone (18.12.2021)	linezolid (19.12.2021) meropenem (19.12.2021)	none	death 20.12.2021
222	F/65	14.12.2021	hypertension, Hashimoto, pancreatitis, obesity	14	29	no	fever refractory to > 3 days of antibiotherapy, dyspnea, respiratory insufficiency despite ventilation support	CT: dense, well-circumscribed lesions with halo sign, crazy paving sing	20.12.2021	dexamethasone (15.12.2021)	piperacillin (22.12.2021) vancomycin (24.12.2021) meropenem (26.12.2021)	voriconazole (25.12.2021)	death 06.01.2022 (after transfer to another ICU)
228	F/73	22.12.2021	hypertension	35	70	no	fever refractory to > 3 days of antibiotherapy, dyspnea, respiratory insufficien	CT: diffuse reticular and alveolar opacities, nonspecific infltrates and consolidat	22.12.2021	dexamethasone (23.12.2021)	vancomycin (27.12.2021) linezolid (29.12.2021) meropenem	none	death 29.12.2021

							cy despite ventilation support	ion, crazy paving sign			(29.12.2021)		
235	M/56	13.12.2021	hypertension, cardiac arrest	9	29	no	dyspnea, respiratory insufficiency despite ventilation support	X-ray: diffuse reticular and alveolar opacities, nonspecifc infltrates and consolidation	13.12.2021	dexamethasone (01.01.2022)	piperacillin (18.12.2021) linezolid (25.12.2021) meropenem (25.12.2021) vankomycin (30.12.2021) colistin (07.01.2022) tigecycline (28.01.2022)	fluconazole (13.12.2021) voriconazole (12.01.2022), caspofungin (27.01.2022)	alive (discharge 09.03.2022)
239	M/73	05.01.2022	leukemia with chemotherapy, diabetes mellitus, hypertension, chronic obstructive pulmonary disease, atherosclerosis	26	72	yes (last cycle of chemotherapy 08.2021)	fever refractory to > 3 days of antibiotherapy, dyspnea, respiratory insufficiency despite ventilation support	X-ray: diffuse reticular and alveolar opacities, nonspecifc infltrates and consolidation	05.01.2022	dexamethasone (05.01.2022)	colistin (10.01.2022) meropenem (10.01.2022)	fluconazole (07.01.2022), voriconazole (15.01.2022)	death 16.01.2022

248	M/64	10.01.2022	none	19	59	no	fever refractory to > 3 days of antibiotherapy, dyspnea, respiratory insufficiency despite ventilation support	CT: nonspecific infltrates and consolidation, crazy paving sign	10.01.2022	dexamethasone (11.01.2022)	meropenem (17.01.2022) colistin (19.01.2022) vancomycin (28.01.2022)	none	death 30.01.2022
254	M/65	15.01.2022	stroke, critical limb ischaemia	14	42	no	dyspnea, respiratory insufficiency despite ventilation support	CT: diffuse reticular and alveolar opacities, nonspecific infltrates and consolidation, crazy paving sign	15.01.2022	dexamethasone (16.01.2022)	linezolid (24.01.2022) meropenem (24.01.2022) cefepim (26.01.2022) colistin (09.02.2022)	voriconazole (24.01.2022)	death 31.03.2022 (after transfer to palliative ward)
14097	F/73	29.10.2021	chronic kidney disease, bladder neoplasm (2019), pulmonary neoplasm (2016), diabetes mellitus, hypertension, hypercholesterolemia,	29	98	yes (during intravesical chemotherapy)	dyspnea, respiratory insufficiency despite ventilation support	X-ray: diffuse reticular and alveolar opacities, nonspecific infltrates and consolidation, Wedge-	29.10.2021	dexamethasone (30.10.2021)	ceftriaxone (23.05.2021) levofloxacin (23.05.2021) linezolid (02.06.2021)	none	death 12.11.2021

			coronary artery disease					shaped infiltrate			colistin (04.06.2021) meropenem (04.06.2021)		

F – female, M – male

Table S2. The summary of mycological tests results in patients with CAPA and the bacterial pathogens isolated from lower respiratory tract and blood

Patient number	*Aspergillus* culture from lower respiratory tract							Other fungi from *Aspergillus* positive culture sample	Bacteria culture from lower respirator tract	Blood culture result	Galactomannan	Mannan	Beta-D-glucan	2020 ECMM/ISHAM case definitione for CAPA[17]	BM-AspICU case definition for IPA[16]
	Clinical sample	Sample collection	Final result	Species	AMB MIC [mg/L]	ITR MIC [mg/L]	VCZ MIC [mg/L]								
57	NBL	09.06.2021	16.06.2021	*Aspergillus fumigatus*	0.19	0.75	0.25	*Candida albicans*	28.05.2021, NBL (negative)<br>01.06.2021, NBL (*Acinetobacter baumannii*)<br>05.06.2021, NBL (*Acinetobacter baumannii*)<br>09.06.2021, NBL (negative)	28.05.2021 (negative)<br>01.06.2021 (negative)<br>05.06.2021 (negative)<br>09.06.2021 (negative)<br>13.06.2021 (negative)	16.06.2021 (serum index ≤0.5)	NT	16.06.2021 (serum 85.03 pg/mL)	Possible	Possible aspergillosis or *Aspergillus* colonisation

									13.06.2021, NBL (negative)						
104	NBL	15.09.2021	22.09.2021	*Aspergillus fumigatus*	0.094	0.38	0.125	*Candida albicans*	15.09.2021, NBL (negative) 19.09.2021, NBL (negative) 21.09.2021, NBL (negative) 23.09.2021, NBL (*Acinetobacter baumannii*)	15.09.2021 (*Staphylococcus hominis*, *Staphylococcus haemolyticus*) 19.09.2021 (negative) 23.09.2021 (negative)	NT	NT	NT	Possible	Possible aspergillosis or *Aspergillus* colonisation
132	BAL	30.10.2021	10.11.2021	*Aspergillus fumigatus*	0.047	0.5	0.032	*Candida albicans*	30.10.2021, BAL (*Staphylococcus aureus* MRSA)	30.10.2021 (negative) 10.11.2021 (*Staphylococcus aureus* MRSA)	30.11.2021 (serum index ≤0.5) 07.12.2021 (serum index ≤0.5)	NT	30.11.2021 (serum 467.22 pg/mL) 07.12.2021 (serum 241.11 pg/mL)	Probable	Possible aspergillosis or *Aspergillus* colonisation
150	NBL	04.11.2021	12.11.2021	*Aspergillus fumigatus*	NT	NT	NT	*Candida albicans*	31.10.2021, NBL (negative) 04.11.2021, NBL (negative)	01.11.2021 (*Staphylococcus hominis*) 04.11.2021 (*Enterococcus faecium* VRE)	NT	NT	NT	Possible	Possible aspergillosis or *Aspergillus* colonisation

181	NBL	26.11.2021	04.12.2021	*Aspergillus fumigatus*	0.125	0.5	0.94	*Candida albicans*	24.11.2021, NBL (negative) 26.11.2021, NBL (negative) 28.11.2021, NBL (negative)	22.11.2021 (negative) 26.11.2021 (negative) 28.11.2021 (negative)	NT	NT	NT	Possible	Possible aspergillosis or *Aspergillus* colonisation
182	NBL	27.11.2021	02.12.2021	*Aspergillus niger*	NT	NT	NT	*Candida albicans*	21.11.2021, NBL (negative) 27.11.2021, NBL (*Acinetobacter baumannii/Klebsiella oxytoca* ESBL)	21.11.2021 (negative), 27.11.2021 (*Acinetobacter baumannii*)	29.11.2021 (serum index ≤0.5)	NT	29.11.2021 (serum 142.41 pg/mL)	Possible	Possible aspergillosis or *Aspergillus* colonisation
187	NBL	28.11.2021	02.12.2021	*Aspergillus fumigatus*	NT	NT	NT	*Candida albicans*	22.11.2021, NBL (negative) 28.11.2021, NBL (*Acinetobacter baumannii)*	22.11.2021 (negative) 28.11.2021 (*Staphylococcus epidermidis*)	28.11.2021 (serum index ≤0.5)	NT	28.11.2021 (serum 191.27 pg/mL)	Possible	Possible aspergillosis or *Aspergillus* colonisation
190	BAL	28.11.2021	02.12.2021	*Aspergillus fumigatus*	0.19	0.75	0.125	*Candida albicans*	28.11.2021, BAL (negative)	28.11.2021 (negative)	20.12.2021 (serum index ≤0.5) (BAL index 0.85)	NT	NT	Probable	Probable
204	NBL	11.12.2021	16.12.2021	*Aspergillus niger*	0.094	1.5	0.094	*Candida albicans*	04.12.2021, NBL (negative) 11.12.2021, NBL (*Acinetobacter baumannii*)	04.12.2021 (*Staphylococcus capitis*) 11.12.2021 (*Candida albicans*)	NT	NT	NT	Possible	Probable

214	NBL	17.12.2021	22.12.2021	*Aspergillus fumigatus*	0.125	0.38	0.125	*Candida tropicalis*	17.12.2021, NBL (*Pseudomonas stutzeri*)	17.12.2021 (negative)	NT	NT	NT	Possible	Possible aspergillosis or *Aspergillus* colonisation
222	NBL	20.12.2021	23.12.2021	*Aspergillus fumigatus*	0.125	0.5	0.094	*Candida albicans*	20.12.2021, NBL (negative) 25.12.2021, NBL (*Staphylococcus aureus* MSSA)	14.12.2021 (negative) 20.12.2021 (negative) 24.12.2021 (*Enterococcus faecalis*)	24.12.2021 (serum index ≤0.5)	24.12.2021 (serum negative: 21.78 pg/mL)	24.12.2021 (serum 111.71 pg/mL)	Possible	Possible aspergillosis or *Aspergillus* colonisation
228	NBL	29.12.2021	03.01.2022	*Aspergillus fumigatus*	0.125	0.35	0.125	*Candida albicans*	22.12.2021, BAL (negative) 29.12.2021, NBL (*Acinetobacter baumannii*)	23.12.2021 (*Enterococcus faecium*, *Staphylococcus hominis*), 24.12.2021 (*Staphylococcus hominis*), 29.12.2021 (*Acinetobacter baumannii*, *Enterococcus faecalis*)	NT	NT	NT	Possible	Possible aspergillosis or *Aspergillus* colonisation

235	NBL	03.01.2022	13.01.2022	*Aspergillus fumigatus*	0.125	0.125	0.125	*Candida krusei*, *Candida inconspicua*	13.12.2021, NBL (negative) 17.12.2021, NBL (negative) 21.12.2021, NBL (negative) 25.12.2021, NBL (negative) 03.01.2022, NBL (negative) 07.01.2022, NBL (negative) 17.01.2022, NBL (negative) 27.01.2022, NBL (negative)	13.12.2021 17.12.2021 21.12.2021 (*Staphylococcus epidermidis*) 25.12.2021 (*Enterococcus faecium*, *Enterococcus faecalis*) 06.01.2022 (negative) 17.01.2022 (negative)	19.01.2022 (serum index ≤0.5) (NBL index 3.85)	NT	19.01.2022 (serum 156.52 pg/mL)	Possible	Possible aspergillosis or *Aspergillus* colonisation
239	NBL	05.01.2022	13.01.2022	*Aspergillus fumigatus*	0.094	0.5	0.19	*Candida albicans*	05.01.2022, NBL (*Acinetobacter baumannii*) 06.01.2022, NBL (*Acinetobacter baumannii*) 14.01.2022, NBL (*Acinetobacter baumannii*)	05.01.2022 (negative) 06.01.2022 (negative) (*Acinetobacter baumannii*) 11.01.2022 (negative) 14.01.2022 (*Acinetobacter baumannii*, *Staphylococcus epidermidis*)	12.01.2022 (serum index 7.60)	NT	12.01.2022 (serum 523.45 pg/mL)	Probable	Probable
248	NBL	10.01.2022	17.01.2022	*Aspergillus fumigatus*	NT	NT	NT	*Candida albicans*, *Penicillium* sp.	10.01.2022, NBL (negative) 16.01.2022, NBL	10.01.2022 (negative) 16.01.2022 (*Acinetobac*	NT	NT	NT	Possible	Possible aspergillosis or *Aspergillus*

									(*Acinetobacter baumannii*) 17.01.2022, NBL (*Acinetobacter baumannii*) 23.01.2022, NBL (*Acinetobacter baumannii*)	*ter baumannii*) 23.01.2022 (*Enterococcus faecalis*)					colonisation
254	NBL	15.01.2022	21.01.2022	*Aspergillus fumigatus*	0.094	0.125	0.047	*Candida dubliniensis*	16.01.2022, NBL (*Chryseobacterium indologenes*) 20.01.2022, NBL (*Escherichia coli*) 24.01.2022, NBL (*Escherichia coli*)	15.01.2022 (negative) 20.01.2022 (negative) 23.01.2022 (*Escherichia coli*)	NT	NT	NT	Possible	Possible aspergillosis or *Aspergillus* colonisation
14097	NBL cultures negative for *Aspergillus*: 29.10.2021 (fungi negative) 09.11.2021 (*Candida albicans*)								09.11.2021, NBL (K*lebsiella pneumoniae* KPC)	29.10.2021 (negative) 05.11.2021 (negative) 09.11.2021 (negative)	10.11.2021 (serum index 8.48)	10.11.2021 (serum 24.15 pg/mL)	10.11.2021 (serum 523.45 pg/mL)	Probable	Probable

NBL - non-bronchoscopic lavage, BAL – bronchoalveolar lavage, AMB - amphotericin B, ITR - itraconazole, VCZ - voriconazole, NT – not tested, MIC – minimal inhibitory concentration [mg/L]
